# Exploring the Development of Wild Microbiomes in the Eastern Fence Lizard

**DOI:** 10.1111/1758-2229.70373

**Published:** 2026-07-28

**Authors:** Michael S. Grapin, Justin Wright, Jeremy Chen See, Samantha Anderson, Regina Lamendella, John Matter

**Affiliations:** ^1^ Biology, Juniata College Huntingdon Pennsylvania USA; ^2^ Wright Labs LLC Huntingdon Pennsylvania USA

**Keywords:** 16S rRNA gene, bacterial succession, gut microbiome, Sceloporus, wild microbiome

## Abstract

This study examines the gut microbiome from faecal samples of a reptilian model ( *Sceloporus undulatus* ). The dynamic host‐symbiote interactions between gut microbiota are essential in metabolic processes and overall health of the host organism. We used 16S rRNA gene sequencing to profile the bacterial community from the guts of wild Eastern Fence Lizards. Microbiome profiles were collected at four distinct time points when lizards were actively foraging in the environment and across four age groups, aiming to characterise the natural variation in bacterial diversity. Environmental bacteria such as *Mycobacterium* and *Pantoea* in young of year (YOY) lizards underwent successional microbiome changes to taxa including *Caproiciproducens*, *Eubacterium*, *Fusobacterium* and *Roseburia* in adults, reflecting dietary shifts from maternal nutrients and early‐stage prey to fibre‐rich arthropod diets. Juvenile lizards exhibited a simple gut microbiome dominated by pioneer colonisers (Firmicutes, Bacteroidota and Proteobacteria), with alpha diversity increasing rapidly within a month to near adult levels. This research extends our understanding of reptilian microbiomes, capturing a snapshot of the ecological complexities of Eastern Fence Lizard microbiomes. As wild lizards mature, their gut microbiome becomes more stable and resilient to environmental disturbances, developing a diverse and balanced bacterial community that supports functional redundancy and ecosystem stability.

## Graphical abstract

We used 16S rRNA gene sequencing to profile the bacterial community from the guts of wild Eastern Fence Lizards. Microbiome profiles were collected at four distinct time points when lizards were actively foraging in the environment and across four age groups, aiming to characterise the natural variation in bacterial diversity. As wild lizards mature, their gut microbiome becomes more stable and resilient to environmental disturbances, developing a diverse and balanced bacterial community that supports functional redundancy and ecosystem stability.

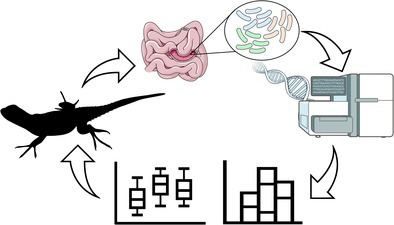

## 1 Background

Microbiomes are intricate communities of microorganisms living within and around organisms and play crucial roles in various aspects of biology, including health, ecology and evolution. These bacterial communities interact with their host organisms in complex ways, influencing digestion, immunity and even behaviour. Furthermore, microbiomes impact ecosystem dynamics, nutrient cycling and disease susceptibility across diverse environments (McFall‐Ngai et al. 2013) and are directly intertwined with organismal ecology and evolution. Specifically, the vertebrate gut microbiome has been implicated in affecting host nutrition, growth, disease and reproductive fitness (Colston and Jackson 2016; Ley et al. 2008). Extensive studies have profiled mammalian microbiomes (Bleich and Fox 2015; Proctor et al. 2019; Turnbaugh et al. 2007) and other model organisms; less is known about the diversity and unique roles of microbiomes within reptilian branches of the tree of life.

Research has shown that reptilian microbiomes undergo constant fluctuations influenced by factors such as diet, environmental conditions, host physiology and interactions with other organisms. For reptiles, whose habitats and diets can vary widely, these fluctuations in the microbiome play essential roles in maintaining health, aiding digestion and potentially even defending against pathogens. Understanding the dynamics of reptilian microbiomes not only sheds light on the intricacies of reptile biology but also offers insights into broader ecological and evolutionary processes (Vasconcelos et al. 2023), diet (Jiang et al. 2017; Lemieux‐Labonté et al. 2022; Montoya‐Ciriaco et al. 2020) and environmental conditions (Liu et al. 2022) have been implicated in shaping lizard bacterial communities. Intrinsic factors such as reproductive events (Trevelline et al. 2019), evolutionary relatedness (Bunker and Weiss 2022; Hong et al. 2011) and bacterial localisation (Kohl et al. 2017; Smith et al. 2021) have also been shown to alter bacterial ecology. Although individual elements play a role, the structure of the gut microbiome is ultimately influenced by a combination of multiple factors (Colston 2017). The role of age in shaping the microbiome is still not entirely understood. From mammalian studies, the microbiome develops when bacteria colonise their host (Faith et al. 2013). However, within lizards, this maturation process has not been fully described.

We present the faecal bacterial community profiles from Eastern Fence Lizard ( *Sceloporus undulatus* ), a medium‐sized phrynosomatid lizard found throughout the central and eastern United States. This species lives in forest edge habitats and open and disturbed mixed hardwood ecosystems and is an insectivorous generalist with respect to dietary preference. Sampling of wild microbiomes is essential to understanding the complex ecological networks of microbes (Hird 2017). Utilising 16S rRNA gene Illumina‐tag sequencing, our study delves into the intricacies of the gut microbiome of *Sceloporus undulatus* , offering a comprehensive exploration of bacterial communities at various stages of development throughout the maturation process of wild lizards. By employing 16S rRNA gene sequencing, the study characterises and compares the composition and diversity of gut bacterial communities across different age groups of Eastern Fence Lizards, shedding light on how the microbiome shifts throughout their development. This research provides valuable insights into the dynamics of lizard‐microbiome interactions and their potential implications for lizard health, ecology and evolution.

## 2 Materials and Methods

### 2.1 Sample Collection

Lizards were collected from an established study site adjacent to Raystown Lake (Huntingdon Co., Pennsylvania, USA). The study site is a shale barren location with a unique ecology and a long history of lizard population assessment. Lizards were caught by hand or by a slip noose made of dental floss suspended from a collapsible rod. In the field, animals were weighed to the nearest 0.1 g and their snout‐vent length (SVL) was measured to the nearest millimetre. Lizards were then kept in ethanol‐sterilised plastic containers and returned to the lab overnight to provide time for them to produce a faecal specimen. Sampling was repeated at four periods important to the lizard life cycle. (1) April–May (proximate to emergence from brumation); *n* = 40, (2) June (includes peak reproductive activity); *n* = 43, (3) July–August (post‐reproductive period); *n* = 34 and (4) September–October (includes hatchlings [young‐of‐the‐year; YOY] and adults in preparation for over‐winter dormancy); *n* = 49. From each sampling period lizards were classified into their respective age groups and sorted into five groups. Adults were animals that had reached sexual maturity at the time of capture. Rising adults were individuals who had hatched the previous year but had not yet been predicted to participate in a reproductive event (i.e., they had not attained reproductive size during the period of reproductive activity). Female lizards in the study population annually produce two clutches of eggs. A first clutch is produced in early June, whilst a second clutch is released approximately 4 weeks later (early July). Both clutches require 60 days for embryonic development. This results in hatchlings entering the population in August (YOY1) and September (YOY2). Young of the year one (YOY1) were the first clutch hatchlings, and young of the year two (YOY2) were second clutch hatchlings. The animal study was reviewed and approved by Juniata's IACUC committee (IACUC#2021–10‐002).

### 2.2 16S rRNA Gene Sequencing of Lizard Faecal Specimens

DNA was extracted from 164 faecal samples using the Zymobiomics DNA Extraction Kit (Orange, California) following the manufacturer's instructions. All 16S rRNA Illumina‐tag PCR reactions were performed on the V4 hypervariable region per the Earth Microbiome Project's protocol (Caporaso et al. 2018) using a T100 Thermal Cycler (Bio‐Rad, Hercules, California, USA) with the following cycling conditions: 94°C 3 min; 35× cycle of 94°C 45 s; 50°C 1 min; 72°C 1 min 30 s; then final extension 72°C for 10 min, hold at 4°C. PCR products were pooled, and gel purified on a 2% agarose gel using the QIAquick Gel Purification Kit (Qiagen, Frederick, Maryland). Prior to sequencing, the purified pool was quality‐checked using a Qubit 4 Fluorometer with 1X dsDNA High Sensitivity Assay (ThermoFisher Scientific, Waltham, Massachusetts, USA) in conjunction with an Agilent 2100 BioAnalyzer and Agilent DNA High Sensitivity DNA kit (Agilent Technologies, Santa Clara, California, USA). The purified pool was stored at −20°C and then sequenced at Wright Labs LLC on an Illumina MiSeq platform using v2 chemistry to produce paired‐end 250 base pair reads.

### 2.3 Bioinformatic and Statistical Analyses

Forward sequences were trimmed at a length of 238 bp and reverse sequences were trimmed at a length of 219 bp. Additional quality filtration was performed at an average expected error of 1, and DADA2 was used to group the remaining sequences into Amplicon Sequence Variants (ASVs) (Callahan et al. 2016; Bolyen et al. 2019). ASV's identified as mitochondria or chloroplasts were removed from the dataset. Samples containing less than 1000 sequences were filtered out of the dataset due to poor coverage, resulting in analysis of 163 samples collected in this study. The quality‐filtered reads were analysed using the QIIME 2.0 software package (Bolyen et al. 2019). ASV taxonomy was assigned using a Naive Bayes classifier with taxonomy assigned using the Silva database (Quast et al. 2013). Adjusted *p* values (denoted as *q* values) using the Hockenberg method (Benjamini and Hochberg 1995) to correct for false discovery rate were considered significant below a 0.05 alpha threshold.

Alpha diversity metrics were constructed from the ASV table and rarefied to a maximum depth of 1500. A rooted phylogenetic tree was created using the ASV representative sequences aligned using MAFFT (Katoh and Standley 2013) and the tree was developed based on that alignment using FastTree 2 (Price et al. 2010). Rarefaction curves were constructed to demonstrate alpha diversity values approaching a horizontal asymptote indicative of adequate depth (Figure S1). Kruskal–Wallis pairwise comparisons were performed and analyses of age groups were reported based on observed features and Faith's phylogenetic diversity (Faith 1992).

The ASV table was normalised using metagenomeSeq's Cumulative Sum Scaling (CSS) algorithm to account for between‐sample differences in sequencing effectiveness (Paulson 2013). Beta diversity metrics were constructed from the normalised ASV table and rooted phylogenetic tree artefact. Weighted Unifrac distance matrices (Chen et al. 2012) were calculated using QIIME2. Permutational Multivariate Analysis of Variance (PERMANOVA) comparisons were used to determine if significant differences exist based on age groups.

Linear Discriminant Analysis Effect Size (LEfSe) (Segata et al. 2011) was performed to determine if bacteria taxa were more abundant in certain age groups. LEfSe analysis was conducted with the filtered ASV table after it underwent Counts per million (CPM) normalisation. Taxa were summarised at the species level, and a Linear Discriminant Analysis (LDA) cutoff was set at 2. Differential abundant taxa were statistically significant at a *p*‐value cut‐off of 0.05.

Partial Least Squares Discriminant Analysis (PLS‐DA) was done with the mixOmics R package (Rohart et al. 2017), and the resulting model was validated using 10‐fold cross‐validation and 10 iterations. Classification error plots can be found in Supporting Information (Figure S2).

Core microbiome analysis was conducted using the Qiime2 core features plugin. A threshold of 75% of samples having a recurrent feature was determined as a core feature (Neu et al. 2021).

## 3 Results

### 3.1 Sequencing Statistics

Illumina sequencing generated 4,206,027 total sequences containing an average frequency of 24,741 sequences for both forward and reverse reads across our 164 samples. DADA2 retained 73% merged and denoised sequences per sample. Samples containing less than 1000 sequences were filtered out of the dataset due to poor coverage, resulting in analysis of 163 samples for downstream analysis. These samples comprised 2,991,108 total sequences, encompassing 1109 unique ASVs.

### 3.2 Core Microbiome

**FIGURE 1:**
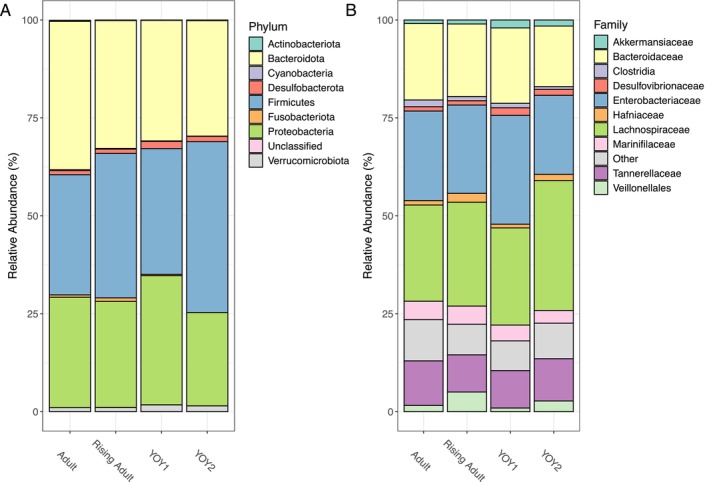
Relative Abundance of Taxonomic Families Summarised by *Sceloporus* Age Class. Stacked bar charts show the top 10 most abundant phylums (A) and families (B) between the four age classes. Resampled YOY1 were condensed into one group to display, and the remaining brackets included all time points. Taxonomic families outside the top 10 are included in the ‘other’ category, as seen in grey.

At the family taxonomic level, *Lachnospiracea, Enterobacteriaceae* and *Bacteriodaceae* were the most abundant bacterial families across all samples. These three taxa comprised 68% of the bacterial community for each age group. The relative abundance of bacterial taxa within the four age cohorts is consistent with bacterial taxa found in the lizard gut core microbiome (Figure 1). All other taxonomic families termed ‘other’ in Figure 1 consisted of an average of 8.7%. Comparisons between adults and rising adults showed there were 12 taxonomic families considered core, whilst including YOY only resulted in 5 core families. The additional families were *Marinifilaceae, Clostridia, Oscillospiraceae, Peptococcaceae, Ruminococcaceae, Rikenellaceae, Erysipelotrichaceae, Selenomonadaceae, Eggerthellaceae, Butyricicoccaceae, Veillonellales‐Selenomonadales, Sporomusaceae* and *Anaerovoracaceae*. Summarising findings can be found below (Table 1).

**TABLE 1:** Core microbiome taxonomic comparisons at the family level.

Group comparison	Percent of samples	Count	Taxa
Adult and rising adult	75%	12	*Lachnospiraceae, Enterobacteriaceae, Bacteroidaceae, Tannerellaceae, Marinifilaceae, Clostridia, Oscillospiraceae, Peptococcaceae, Ruminococcaceae, Rikenellaceae, Erysipelotrichaceae, Selenomonadaceae, Eggerthellaceae, Butyricicoccaceae, Veillonellales‐Selenomonadales, Sporomusaceae, Anaerovoracaceae*
Adult, rising adult, YOY	75%	5	*Lachnospiraceae, Bacteroidaceae, Enterobacteriaceae, Tannerellaceae, Ruminococcaceae*

**TABLE 2:** Alpha diversity *q*‐value table for significant observed features pairwise comparisons.

Group 1	Group 2	*q*‐value
August YOY1 (*n* = 10)	September YOY1 (*n* = 12)	0.016
August YOY1 (*n* = 10)	August adult (*n* = 12)	0.016
August YOY1 (*n* = 10)	June adult (*n* = 25)	0.016
August YOY1 (*n* = 10)	May adult (*n* = 26)	0.017
August YOY1 (*n* = 10)	September adult (*n* = 13)	0.017
August YOY1 (*n* = 10)	May rising adult (*n* = 14)	0.023
August rising adult (*n* = 12)	September YOY1 (*n* = 12)	0.020
August rising adult (*n* = 12)	June adult (*n* = 25)	0.029
August rising adult (*n* = 12)	September adult (*n* = 13)	0.035
August rising adult (*n* = 12)	May adult (*n* = 26)	0.038
August adult (*n* = 12)	August rising adult (*n* = 12)	0.021

### 3.3 Alpha Diversity of Gut Microbiota Across Age Groups

**FIGURE 2:**
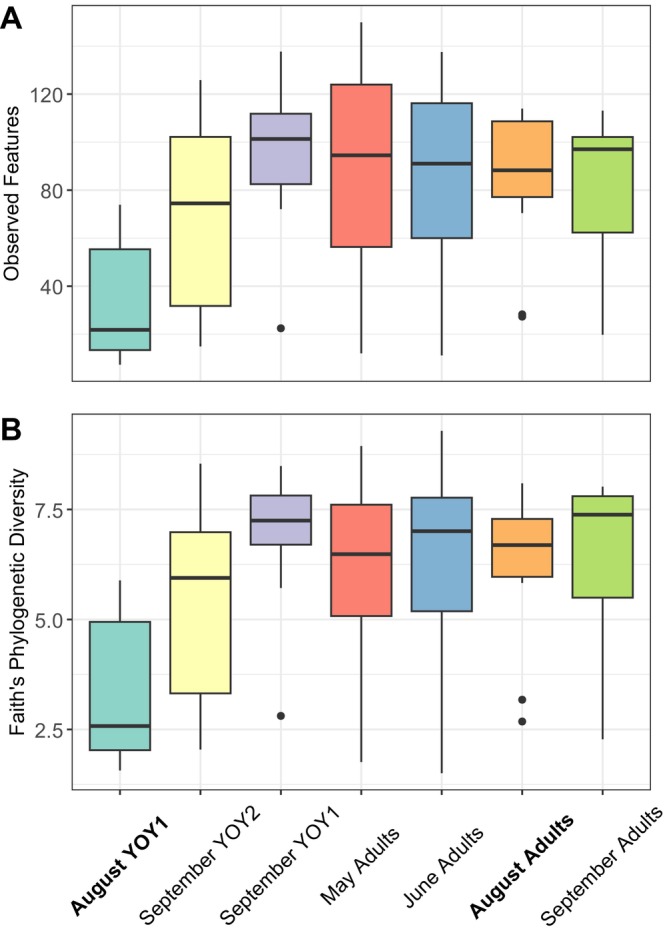
Alpha diversity of gut bacterial communities in *Sceloporus*. (A) Box plot of observed features (ASVs) from YOY cohorts and Adult cohorts at these longitudinal time points from youngest age to oldest. (B) Box plot of Faith's phylogenetic diversity from YOY and Adult gut bacterial communities. Observed features and Faith's phylogenetic diversity metrics were calculated on a rarefied ASV table. Both observed features and Faith's phylogenetic diversity comparisons were evaluated for significance using Kruskal–Wallis tests, and all bolded labels represent significant *q* values below the 0.05 threshold. A full list of significant *q* values can be found in Tables 2 and 3.

Alpha diversity analysis of observed features revealed a significant difference when comparing across all age groups (Kruskal–Wallis; *p* = 0.002), with younger lizards having fewer observed features (unique ASVs) than adult lizards (Figure 2A, Table S1). The August YOY1 group had the least observed features, with a median of 21, ranging from 7 to 73 features. When the same age cohort was sampled again (September YOY1) we observed a median value of 101 features, with 22–137 features (Kruskal–Wallis; *q* = 0.016, Table S3). Contrastingly, no significant difference was observed in the comparisons between the YOY1 and YOY2 cohorts. It should be noted that August YOY1 had significantly lower observed features than all adult cohorts (Table S1). The rising adult cohort had a comparable number of median features to adults, yet this cohort also had the most variability compared to all other cohorts.

The analysis of phylogenetic diversity significantly differed across age groups (Kruskal–Wallis; *p* = 0.002). Adult cohorts consistently displayed higher bacterial diversity, with median values ranging from 6.4 to 7.3 (Figure 2B, Table S2). In contrast, Young‐of‐Year (YOY) groups showed a marked increase in diversity from August YOY1 (median = 2.57) to September YOY1 (median = 7.24). Pairwise comparisons revealed that August YOY1 had significantly lower bacterial diversity than the adult groups (Table 3). However, there were no significant differences in diversity between the YOY1 and YOY2 groups (Table S4). Additionally, a significant increase in diversity was observed between the August and September YOY1 groups (*q* = 0.015), suggesting changes within the same cohort over time. The August Rising Adults group had notably lower median diversity (median = 2.91) and displayed significant differences compared to both adult and YOY groups (Table 3).

**TABLE 3:** Alpha diversity *q*‐value table for significant Faith's phylogenetic diversity pairwise comparisons.

Group 1	Group 2	*q*‐value
August YOY1 (*n* = 10)	September YOY1 (*n* = 12)	0.015
August YOY1 (*n* = 10)	August adult (*n* = 12)	0.018
August YOY1 (*n* = 10)	June adult (*n* = 25)	0.018
August YOY1 (*n* = 10)	May adult (*n* = 26)	0.018
August YOY1 (*n* = 10)	September adult (*n* = 13)	0.018
August rising adult (*n* = 12)	September YOY1 (*n* = 12)	0.018
August YOY1 (*n* = 10)	May rising adult (*n* = 14)	0.018
August rising adult (*n* = 12)	June adult (*n* = 25)	0.040
August adult (*n* = 12)	August rising adult (*n* = 12)	0.040
August rising adult (*n* = 12)	September adult (*n* = 13)	0.049

### 3.4 Beta Diversity: Comparisons Between Age Groups

**FIGURE 3:**
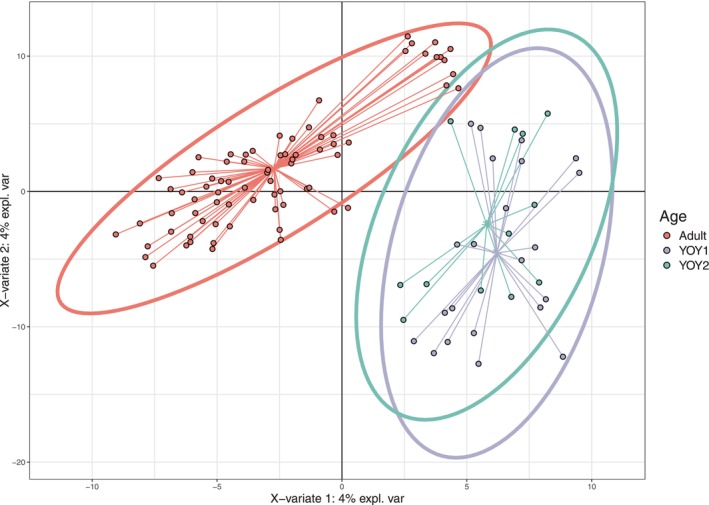
Partial least squares discriminant analysis (PLS‐DA) percentage explained variation of bacterial communities between Adults, YOY1 hatchlings and YOY2 hatchlings. Plotted values are from a normalised taxonomy table using the PLS‐DA method to compare the variation of microbiomes between Adult, YOY1 and YOY2 aged lizards. Ellispe identify borders between‐group comparisons and lines to points show distance to the centroid. The X and Y axes explain the percent variation between age classes.

Beta diversity significantly differed amongst the age groups (PERMANOVA; *p*‐value = 0.001). The most substantial source of variation was observed when a YOY cohort was compared to other sampled groups (Table 4). Distinguishing age groups by their respective microbiome illustrates a clear distinction between Adult and YOY individuals, with both the X and Y axis predictors explaining 4% of the variation (Figure 3). However, when comparing YOY1 and YOY2, there is substantial overlap, indicating slight variation between the age cohorts compared to adult lizards. Error rates for the PLS‐DA model were approximately 30%, suggesting that 70% of the time, observed variation can help explain differences in bacterial communities between adult and YOY lizards (Figure S2). This indicates a reasonably effective model for discriminating between the microbiomes of adult and young of the year lizards based on the provided predictor variables.

**TABLE 4:** Pairwise Permanova results with significant *q*‐values between age classes.

Group 1	Group 2	Pseudo‐F	*q*‐value
August YOY1	June adult	5.130	0.044
August YOY1	September YOY1	6.260	0.044
August YOY1	September adult	4.547	0.048
August YOY1	May rising adult	4.972	0.048
August rising adult	September YOY1	8.685	0.044
August rising adult	September adult	5.127	0.044
August rising adult	May adult	4.540	0.047
August rising adult	May rising adult	5.915	0.047
August adults	August rising adult	5.238	0.044

### 3.5 Differentially‐Abundant Bacterial Taxa

**FIGURE 4:**
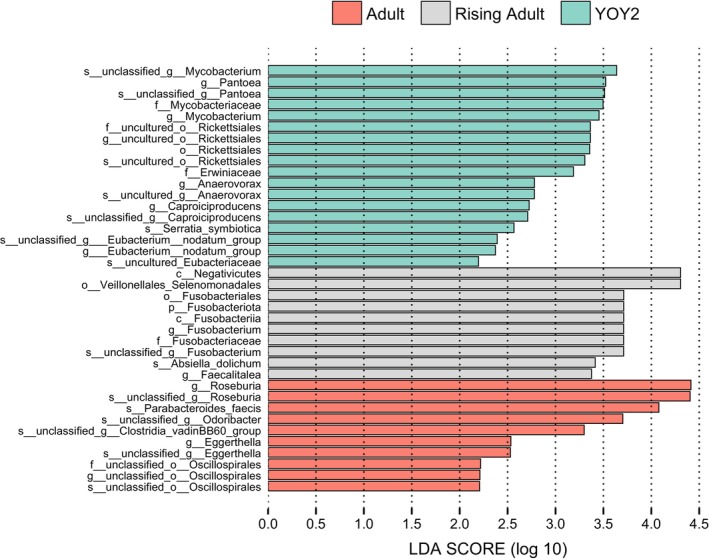
LeFSe shows enriched bacterial taxa between adults, rising adults, YOY1 and YOY2. Plotted taxa are from a minimum LDA score cut‐off of 2 and have unique phylogenetic classification. No taxa were found to be differentially abundant in the YOY1.

Significantly differentially‐abundant (LDA > 2.0, *p* < 0.05) bacterial taxa were observed between each age group using LeFSe analysis, and 38 taxonomic bacterial classifications were enriched amongst the different age groups (Figure 4). YOY2 has the most enriched taxa, having 18 classifications belonging to seven different orders. Rising adults had 10 enriched taxa amongst three different orders. Adult lizards have 10 enriched taxa across three orders. YOY1 was found to have no differentially abundant bacteria. Amongst taxa found at YOY2, there were multiple classifications in *Mycobacteriaceae, Rickettsiales, Caprociciproducens* and *Eubacterium*. Rising adults had limited phylogenetic resolution, but taxa were classified as multiple *Veillonella* and *Fusobacterium* species. Adults had taxa within the genus *Roseburia* in addition to other commonly found anaerobes to the gut microbiome.

## 4 Discussion

This study leveraged 16S rRNA gene sequencing to describe the development of wild microbiomes from Eastern Fence Lizards, *Sceloporus undulatus*. Successional changes in the gut microbiome of lizards from the juvenile stage to adulthood involve dynamic shifts in bacterial composition, diversity and functional capacity. Our study took an exploratory approach to capture this bacterial diversity and explain trends in the context of fence lizards' life‐history ecology.

In the early stages of development, juvenile lizards typically exhibit a simple gut microbiome dominated by pioneer colonisers (Lee et al. 2023; Milani et al. 2017; van Best et al. 2015). Alpha diversity results revealed notable differences in bacterial community structure, with newly hatched YOY lizards displaying lower diversity than adults (Figure 2). However, within about a month, the microbiome of YOY lizards diversified to near adult‐level diversity. Establishing a diverse and balanced bacterial community is crucial for providing functional redundancy and ecosystem stability, enabling the gut microbiome to maintain its composition and function under varying conditions. Like other vertebrates, we observed core microbiota in Eastern Fence Lizards, primarily consisting of Firmicutes, Bacteroidota and Proteobacteria (Figure 1). As juvenile lizards transition from a diet of maternal nutrients (abdominal yolk) and early‐stage prey to more diverse adult diets, a corresponding shift in gut microbiome composition occurs.

The observed developmental patterns in bacterial composition align with findings in other vertebrates. For example, microbiome maturation typically begins with an initial increase in diversity in newborns, followed by stabilisation in adulthood, a trend mirrored in human microbiome development (Bäckhed et al. 2015). Initial exposure to colonising microbes is foundational in shaping bacterial community development (Milani et al. 2017). Our observations in wild lizards reflect the presence of these core taxa, with the metabolic potential for maintaining a balanced microbiome.

The faster timescale of microbiome maturation in lizards compared to mammals may be attributed to multiple factors. One key aspect is their shorter intestinal tracts, which likely facilitate a more rapid establishment of bacterial homeostasis. Shorter gut lengths correlate with faster transit times and reduced microbial residence time, thereby selecting bacterial taxa capable of rapid colonisation and adaptation (Stevens and Hume 1998; McWhorter et al. 2009). Evidence suggests that gut size influences microbiome diversity, with larger gastrointestinal tracts supporting greater microbial richness and functional capacity (Godon et al. 2016; Karasov et al. 2011). In lizards, the small size of the gastrointestinal tract may impose constraints on the microbiome's carrying capacity, limiting both the overall abundance of microorganisms and the availability of specific ecological niches. Additionally, smaller body size is closely tied to an organism's lifespan and growth rate (Angilletta Jr et al. 2004; Ringsby et al. 2015; Savage et al. 2004). Lizards exhibit accelerated developmental timelines, with young individuals reaching adulthood in 18–24 months. This rapid pace of development may drive microbiome maturation over a compressed timeframe, potentially favouring bacterial taxa foundational to gut microbiome structure and function. Such foundational taxa often play critical roles in host physiology, including nutrient absorption, immune modulation and pathogen resistance, and may reflect long‐standing symbiotic relationships (Hooper and Gordon 2001; Shapira 2016).

Environmental exposure is another critical factor influencing the diversification of the gut microbiome in young lizards. YOY (young‐of‐year) lizards undergo a swift nutritional transition from yolk‐based sustenance to an insectivorous diet within a few weeks of hatching. This dietary shift likely induces substantial changes in gut microbiome composition and functionality, as demonstrated in other vertebrates undergoing dietary transitions (Kohl and Dearing 2012; David et al. 2014). The smaller prey items accessible to YOY lizards further shape their microbiome's trajectory. Due to their limited gape width and smaller body size, YOY lizards are constrained to consume smaller, less nutritionally complex prey items, which may result in a diet lower in microbial diversity and functional breadth than adults. This dietary bottleneck could influence the rate and direction of microbiome development, selecting bacterial taxa that thrive under such resource‐limited conditions (Hawlena et al. 2010; Kohl et al. 2015).

Moreover, lizards' reliance on environmental sources for bacterial inoculation, such as soil, plants and prey, underscores the importance of ecological interactions in shaping gut microbial communities. Variability in habitat types, prey availability and environmental microbiota may contribute to interspecific differences in microbiome composition and maturation rates amongst lizards (Brucker and Bordenstein 2013). These factors highlight the dynamic interplay between host physiology, life history and environmental influences driving the rapid microbiome maturation observed in lizards. Future studies examining how these factors influence lizards' microbial community assembly and functionality could provide broader insights into vertebrate microbiome evolution and ecology.

It is known that core taxa in the phyla Firmicutes, Bacteroidota, Proteobacteria and Actinobacteria are dominant taxa in gut microbiomes (Fujisaka et al. 2023). These bacteria have been selected for the gut ecosystem because of their functional roles in regulating metabolism. This redundancy is evolutionarily conserved and is seen across both reptilian and other vertebrate models (McFall‐Ngai et al. 2013; Ley et al. 2008). Our findings align with previous studies on lizard gut microbiomes, which reported similar bacterial phyla distributions (Alemany et al. 2022; Zhou et al. 2020). Amongst these, the analysis of *Sceloporus* showed the greatest concordance with our data (Bunker et al. 2022). Specifically, the most abundant taxa identified in both our study and others include *Lachnospiraceae*, *Enterobacteriaceae*, *Bacteroidaceae*, *Akkermansiaceae*, *Desulfovibrionaceae*, *Marinifilaceae* and *Tannerellaceae*. These shared taxa underscore common bacterial community structures in lizard models, providing robust comparability across studies. Similarly, the core microbiome analysis revealed bacterial taxa belonging to the phyla Firmicutes, Baceroidota and Pseudomodota to which a representative human sample (Falony et al. 2016) saw similar core taxa.

As lizards mature and are exposed to a broader range of environmental microbes and dietary substrates, the gut microbiome rapidly diversifies, reflecting a more complex and stable bacterial community. Young of year lizards transition from a diet primarily composed of maternal nutrients or early‐stage prey items to more diverse adult diets of insect arthropods. Differential abundance analysis poses a narrative of successional changes that occur in gut ecosystems. First, young lizards are seeded heavily by environmental bacteria. Enriched taxa such as *Mycobacterium* and *Pantoae* are commonly found in soil (Janssen 2006; Walterson and Stavrinides 2015), water (Walterson and Stavrinides 2015; Delghandi et al. 2020) and plant material (Walterson and Stavrinides 2015; Tian and Li 2017). Additionally, recently hatched lizards might consume substrates or small prey items that harbour these bacteria. Metabolism of carbohydrates and fibre is critical to sustenance; bacteria like *Caproiciproducens* (Kim et al. 2015) and Eubacterium (Duncan et al. 2007; Dworkin et al. 2006) being enriched suggests metabolism of these early substrates. YOY2 had the most enriched taxa, suggesting their microbiome is still fluctuating. Transitioning to a broadening insectivorous diet, rising adults show taxa that thrive on lactates (Reichardt et al. 2014; Wang et al. 2020) (Negativicutes) from complex carbohydrate fermentation and amino acid‐rich environments (*Fusobacterium*) indicative of protein consumption. As adult lizard microbiomes stabilise, we see taxa representing consistent metabolic pathways. *Roseburia*, known as short‐chain fatty acid (SCFA)‐producing bacteria (Tamanai‐Shacoori et al. 2017) which ferment high fibre substrates, were enriched in adult lizards. The consumption of larger arthropods whose chitin exoskeleton provides a possible source of fibre that bacteria can metabolise into critical energy‐ready SCFAs.

## 5 Conclusions

Our study advances the understanding of microbiome development in a reptilian model, highlighting the dynamic process of bacterial colonisation, diversification and adaptation as lizards mature. Future research should address the limitations of 16S rRNA gene sequencing, which provides taxonomic resolution for bacteria and archaea but cannot capture functional potential or detect non‐bacterial members of the microbiome, such as fungi and single‐celled eukaryotes. These organisms play critical roles in maintaining the complex ecological balance of the microbiome and are integral to a more comprehensive understanding of microbial interactions. Future studies should leverage shotgun metagenomics sequencing to bridge this gap by enabling functional gene profiling and the inclusion of diverse microbial taxa beyond bacteria and archaea. Future investigations should incorporate early microbiome sampling in controlled laboratory environments to refine our understanding of the microbial inoculation processes and identify their primary sources. Additionally, we encourage more studies on wild microbiomes to capture the full diversity of bacterial communities in organisms across ecosystems. Physiological changes during growth, such as shifts in gut pH, mucosal structure and immune function, create niche opportunities for specific bacterial taxa to establish and persist in the gut microbiome. As lizards mature, their gut microbiome becomes more stable and resilient to environmental disturbances, developing a diverse and balanced bacterial community that supports functional redundancy and ecosystem stability. Overall, successional changes in the gut microbiome of lizards from the juvenile stage to adulthood reflect a dynamic process of bacterial colonisation, diversification and adaptation influenced by host development, dietary transitions and environmental factors.

## Author Contributions

**Michael S. Grapin:** conceptualization, investigation, methodology, visualization, formal analysis, writing – review and editing, writing – original draft. **Justin Wright:** writing – review and editing, validation, software, methodology. **Jeremy Chen See:** writing – review and editing, software, validation, methodology. **Samantha Anderson:** writing – review and editing, resources, methodology. **Regina Lamendella:** conceptualization, funding acquisition, writing – review and editing, supervision. **John Matter:** conceptualization, writing – review and editing, resources, supervision.

## Funding

This work was supported by the Juniata College's Student Scholarly Endeavour Committee, Howard Hughes Medical Institute Precollege and Undergraduate Science Education Programme. National Science Foundation (DBI‐1248096).

## Ethics Statement

The authors have nothing to report.

## Consent

The authors have nothing to report.

## Conflicts of Interest

Four of six authors are affiliated with Wright Labs LLC. The other authors declare no conflicts of interest.

## Supporting information


**Figure S1:** Alpha rarefaction curves for alpha diversity analysis.
**Figure S2:** Classification error plots from PSL‐DA analysis.
**Table S1:** Descriptive statistics for figure 2A measuring observed features.
**Table S2:** Descriptive statistics for figure 2B measuring faith's phylogenetic diversity.
**Table S3:** Alpha diversity values table for observed features Kruskal–Wallis pairwise comparisons.
**Table S4:** Alpha diversity values table for faith's phylogenetic diversity Kruskal–Wallis pairwise comparisons.

## Data Availability

The data that support the findings of this study are openly available in NCBI Short Read Archives at https://www.ncbi.nlm.nih.gov/bioproject/PRJNA1215967, reference number PRJNA1215967.

## References

[emi470373-bib-0001] Alemany, I. , A. Pérez‐Cembranos , V. Pérez‐Mellado , et al. 2022. “Faecal Microbiota Divergence in Allopatric Populations of Podarcis Lilfordi and P. Pityusensis, Two Lizard Species Endemic to the Balearic Islands.” Microbial Ecology 85: 10.1007/S00248‐022‐02019‐3. 10.1007/s00248-022-02019-3 PMC10167182 35482107

[emi470373-bib-0002] Angilletta, M. J., Jr. , T. D. Steury , and M. W. Sears . 2004. “Temperature, Growth Rate, and Body Size in Ectotherms: Fitting Pieces of a Life‐History Puzzle1.” Integrative and Comparative Biology 44: 498–509. 21676736 10.1093/icb/44.6.498

[emi470373-bib-0003] Bäckhed, F. , J. Roswall , Y. Peng , et al. 2015. “Dynamics and Stabilization of the Human Gut Microbiome During the First Year of Life.” Cell Host & Microbe 17: 690–703. 25974306 10.1016/j.chom.2015.04.004

[emi470373-bib-0004] Benjamini, Y. , and Y. Hochberg . 1995. “Controlling the False Discovery Rate: A Practical and Powerful Approach to Multiple Testing.” Journal of the Royal Statistical Society. Series B, Statistical Methodology 57: 289–300.

[emi470373-bib-0005] Bleich, A. , and J. G. Fox . 2015. “The Mammalian Microbiome and Its Importance in Laboratory Animal Research.” ILAR Journal 56: 153–158. 26323624 10.1093/ilar/ilv031 PMC4854015

[emi470373-bib-0006] Bolyen, E. , J. R. Rideout , M. R. Dillon , et al. 2019. “Reproducible, Interactive, Scalable and Extensible Microbiome Data Science Using QIIME 2.” Nature Biotechnology 37: 852–857. 10.1038/s41587-019-0209-9 PMC7015180 31341288

[emi470373-bib-0007] Brucker, R. M. , and S. R. Bordenstein . 2013. “The Hologenomic Basis of Speciation: Gut Bacteria Cause Hybrid Lethality in the Genus Nasonia.” Science 341: 667–669. 23868918 10.1126/science.1240659

[emi470373-bib-0008] Bunker, M. , and S. Weiss . 2022. “Cloacal Microbiomes of Sympatric and Allopatric Sceloporus Lizards Vary with Environment and Host Relatedness.” PLoS One 17, no. 12: e0279288. 36548265 10.1371/journal.pone.0279288 PMC9779040

[emi470373-bib-0009] Bunker, M. E. , M. O. Martin , and S. L. Weiss . 2022. “Recovered Microbiome of an Oviparous Lizard Differs Across Gut and Reproductive Tissues, Cloacal Swabs, and Faeces.” Molecular Ecology Resources 22: 1693–1705. 34894079 10.1111/1755-0998.13573

[emi470373-bib-0010] Callahan, B. J. , P. J. McMurdie , M. J. Rosen , A. W. Han , A. J. A. Johnson , and S. Holmes . 2016. “DADA2: High‐Resolution Sample Inference From Illumina Amplicon Data.” Nature Methods 13: 581–583. 10.1038/NMETH.3869. 27214047 PMC4927377

[emi470373-bib-0011] Caporaso, J. G. , G. Ackermann , A. Apprill , et al. 2018. EMP 16S Illumina Amplicon Protocol. protocols.io. 10.17504/protocols.io.nuudeww.

[emi470373-bib-0012] Chen, J. , K. Bittinger , E. S. Charlson , et al. 2012. “Associating Microbiome Composition With Environmental Covariates Using Generalized UniFrac Distances.” Bioinformatics 28: 2106–2113. 22711789 10.1093/bioinformatics/bts342 PMC3413390

[emi470373-bib-0013] Colston, T. 2017. “Gut Microbiome Transmission in Lizards.” Molecular Ecology 26: 972–974. 28239927 10.1111/mec.13987

[emi470373-bib-0014] Colston, T. J. , and C. R. Jackson . 2016. “Microbiome Evolution Along Divergent Branches of the Vertebrate Tree of Life: What Is Known and Unknown.” Molecular Ecology 25: 3776–3800. 27297628 10.1111/mec.13730

[emi470373-bib-0015] David, L. A. , C. F. Maurice , R. N. Carmody , et al. 2014. “Diet Rapidly and Reproducibly Alters the Human Gut Microbiome.” Nature 505: 559–563. 24336217 10.1038/nature12820 PMC3957428

[emi470373-bib-0016] Delghandi, M. R. , K. Waldner , M. El‐Matbouli , and S. Menanteau‐Ledouble . 2020. “Identification *Mycobacterium* spp. in the Natural Water of Two Austrian Rivers.” Microorganisms 8: 1305. 32867056 10.3390/microorganisms8091305 PMC7563569

[emi470373-bib-0017] Duncan, S. H. , A. Belenguer , G. Holtrop , A. M. Johnstone , H. J. Flint , and G. E. Lobley . 2007. “Reduced Dietary Intake of Carbohydrates by Obese Subjects Results in Decreased Concentrations of Butyrate and Butyrate‐Producing Bacteria in Feces.” Applied and Environmental Microbiology 73: 1073–1078. 17189447 10.1128/AEM.02340-06 PMC1828662

[emi470373-bib-0018] Dworkin, M. , S. Falkow , E. Rosenberg , K.‐H. Schleifer , and E. Stackebrandt , eds. 2006. The Prokaryotes: Volume 4: Bacteria: Firmicutes, Cyanobacteria. Springer US.

[emi470373-bib-0019] Faith, D. P. 1992. “Conservation Evaluation and Phylogenetic Diversity.” Biological Conservation 61: 1–10.

[emi470373-bib-0020] Faith, J. J. , J. L. Guruge , M. Charbonneau , et al. 2013. “The Long‐Term Stability of the Human Gut Microbiota.” Science 341: 1237439. 23828941 10.1126/science.1237439 PMC3791589

[emi470373-bib-0021] Falony, G. , M. Joossens , S. Vieira‐Silva , et al. 2016. “Population‐Level Analysis of Gut Microbiome Variation.” Science 352: 560–564. 27126039 10.1126/science.aad3503

[emi470373-bib-0022] Fujisaka, S. , Y. Watanabe , and K. Tobe . 2023. “The Gut Microbiome: A Core Regulator of Metabolism.” Journal of Endocrinology 256: e220111. 36458804 10.1530/JOE-22-0111 PMC9874984

[emi470373-bib-0023] Godon, J.‐J. , P. Arulazhagan , J.‐P. Steyer , and J. Hamelin . 2016. “Vertebrate Bacterial Gut Diversity: Size Also Matters.” BMC Ecology 16: 12. 27008566 10.1186/s12898-016-0071-2 PMC4804487

[emi470373-bib-0024] Hawlena, H. , F. Bashey , and C. M. Lively . 2010. “The Evolution of Spite: Population Structure and Bacteriocin‐Mediated Antagonism in Two Natural Populations of Xenorhabdus Bacteria.” Evolution 64: 3198–3204. 20584073 10.1111/j.1558-5646.2010.01070.x

[emi470373-bib-0025] Hird, S. M. 2017. “Evolutionary Biology Needs Wild Microbiomes.” Frontiers in Microbiology 8: 725. 28487687 10.3389/fmicb.2017.00725 PMC5404107

[emi470373-bib-0026] Hong, P. Y. , E. Wheeler , I. K. Cann , and R. I. Mackie . 2011. “Phylogenetic Analysis of the Fecal Microbial Community in Herbivorous Land and Marine Iguanas of the Galápagos Islands Using 16S rRNA‐Based Pyrosequencing.” ISME Journal 5, no. 9: 1461–1470. 21451584 10.1038/ismej.2011.33 PMC3160690

[emi470373-bib-0027] Hooper, L. V. , and J. I. Gordon . 2001. “Commensal Host‐Bacterial Relationships in the Gut.” Science 292: 1115–1118. 11352068 10.1126/science.1058709

[emi470373-bib-0028] Janssen, P. H. 2006. “Identifying the Dominant Soil Bacterial Taxa in Libraries of 16S rRNA and 16S rRNA Genes.” Applied and Environmental Microbiology 72: 1719–1728. 16517615 10.1128/AEM.72.3.1719-1728.2006 PMC1393246

[emi470373-bib-0029] Jiang, H.‐Y. , J.‐E. Ma , J. Li , et al. 2017. “Diets Alter the Gut Microbiome of Crocodile Lizards.” Frontiers in Microbiology 8: 2073. 29118742 10.3389/fmicb.2017.02073 PMC5660983

[emi470373-bib-0030] Karasov, W. H. , C. M. del Rio , and E. Caviedes‐Vidal . 2011. “Ecological Physiology of Diet and Digestive Systems.” Annual Review of Physiology 73: 69–93. 10.1146/annurev-physiol-012110-142152 21314432

[emi470373-bib-0031] Katoh, K. , and D. M. Standley . 2013. “MAFFT Multiple Sequence Alignment Software Version 7: Improvements in Performance and Usability.” Molecular Biology and Evolution 30: 772–780. 23329690 10.1093/molbev/mst010 PMC3603318

[emi470373-bib-0032] Kim, B.‐C. , B. Seung Jeon , S. Kim , H. Kim , Y. Um , and B.‐I. Sang . 2015. “ *Caproiciproducens* Galactitolivorans gen. nov., sp. nov., a Bacterium Capable of Producing Caproic Acid From Galactitol, Isolated From a Wastewater Treatment Plant.” International Journal of Systematic and Evolutionary Microbiology 65: 4902–4908. 26474980 10.1099/ijsem.0.000665

[emi470373-bib-0033] Kohl, K. D. , A. Brun , M. Magallanes , et al. 2017. “Gut Microbial Ecology of Lizards: Insights Into Diversity in the Wild, Effects of Captivity, Variation Across Gut Regions and Transmission.” Molecular Ecology 26: 1175–1189. 27862531 10.1111/mec.13921

[emi470373-bib-0034] Kohl, K. D. , T. L. Cary , W. H. Karasov , and M. D. Dearing . 2015. “Larval Exposure to Polychlorinated Biphenyl 126 (PCB‐126) Causes Persistent Alteration of the Amphibian Gut Microbiota.” Environmental Toxicology and Chemistry 34: 1113–1118. 25651416 10.1002/etc.2905

[emi470373-bib-0035] Kohl, K. D. , and M. D. Dearing . 2012. “Experience Matters: Prior Exposure to Plant Toxins Enhances Diversity of Gut Microbes in Herbivores.” Ecology Letters 15: 1008–1015. 22715970 10.1111/j.1461-0248.2012.01822.x

[emi470373-bib-0036] Lee, M. D. , A. A. Pedroso , B. Lumpkins , Y. Cho , and J. J. Maurer . 2023. “Pioneer Colonizers: Bacteria That Alter the Chicken Intestinal Morphology and Development of the Microbiota.” Frontiers in Physiology 14: 14. 10.3389/fphys.2023.1139321 PMC10090334 37064908

[emi470373-bib-0037] Lemieux‐Labonté, V. , C. Vigliotti , Z. Tadic , et al. 2022. “Proximate Drivers of Population‐Level Lizard Gut Microbial Diversity: Impacts of Diet, Insularity, and Local Environment.” Microorganisms 10, no. 8: 1550. 36013968 10.3390/microorganisms10081550 PMC9413874

[emi470373-bib-0038] Ley, R. E. , C. A. Lozupone , M. Hamady , R. Knight , and J. I. Gordon . 2008. “Worlds Within Worlds: Evolution of the Vertebrate Gut Microbiota.” Nature Reviews. Microbiology 6: 776–788. 18794915 10.1038/nrmicro1978 PMC2664199

[emi470373-bib-0039] Liu, W. , J. Yang , Y. Meng , et al. 2022. “The Divergent Effects of Moderate Climate Warming on the Gut Microbiota and Energetic State of Cold‐Climate Lizards From Open and Semi‐Closed Microhabitats.” Frontiers in Microbiology 13: 1050750. 36483215 10.3389/fmicb.2022.1050750 PMC9722725

[emi470373-bib-0040] McFall‐Ngai, M. , M. G. Hadfield , T. C. G. Bosch , et al. 2013. “Animals in a Bacterial World, a New Imperative for the Life Sciences.” National Academy of Sciences of the United States of America 110: 3229–3236. 10.1073/pnas.1218525110 PMC3587249 23391737

[emi470373-bib-0041] McWhorter, T. J. , E. Caviedes‐Vidal , and W. H. Karasov . 2009. “The Integration of Digestion and Osmoregulation in the Avian Gut.” Biological Reviews 84: 533–565. 19673857 10.1111/j.1469-185X.2009.00086.x

[emi470373-bib-0042] Milani, C. , S. Duranti , F. Bottacini , et al. 2017. “The First Microbial Colonizers of the Human Gut: Composition, Activities, and Health Implications of the Infant Gut Microbiota.” Microbiology and Molecular Biology Reviews 81: e00036‐17. 10.1128/MMBR.00036-17 PMC5706746 29118049

[emi470373-bib-0043] Montoya‐Ciriaco, N. , S. Gómez‐Acata , L. C. Muñoz‐Arenas , et al. 2020. “Dietary Effects on Gut Microbiota of the Mesquite Lizard *Sceloporus grammicus* (Wiegmann, 1828) Across Different Altitudes.” Microbiome 8: 6. 31980039 10.1186/s40168-020-0783-6 PMC6982387

[emi470373-bib-0044] Neu, A. T. , E. E. Allen , and K. Roy . 2021. “Defining and Quantifying the Core Microbiome: Challenges and Prospects.” Proceedings of the National Academy of Sciences of the United States of America 118: e2104429118. 34862327 10.1073/pnas.2104429118 PMC8713806

[emi470373-bib-0045] Paulson, J. N. 2013. “Differential Abundance Analysis for Microbial Marker‐Gene Surveys.” Nature Methods 10: 1200–1202. 24076764 10.1038/nmeth.2658 PMC4010126

[emi470373-bib-0046] Price, M. N. , P. S. Dehal , and A. P. Arkin . 2010. “FastTree 2—Approximately Maximum‐Likelihood Trees for Large Alignments.” PLoS One 5: e9490. 20224823 10.1371/journal.pone.0009490 PMC2835736

[emi470373-bib-0047] Proctor, L. M. , H. H. Creasy , J. M. Fettweis , et al. 2019. “The Integrative Human Microbiome Project.” Nature 569: 641–648. 31142853 10.1038/s41586-019-1238-8 PMC6784865

[emi470373-bib-0048] Quast, C. , E. Pruesse , P. Yilmaz , et al. 2013. “The SILVA Ribosomal RNA Gene Database Project: Improved Data Processing and Web‐Based Tools.” Nucleic Acids Research 41: D590–D596. 23193283 10.1093/nar/gks1219 PMC3531112

[emi470373-bib-0049] Reichardt, N. , S. H. Duncan , P. Young , et al. 2014. “Phylogenetic Distribution of Three Pathways for Propionate Production Within the Human Gut Microbiota.” ISME Journal 8: 1323–1335. 24553467 10.1038/ismej.2014.14 PMC4030238

[emi470373-bib-0050] Ringsby, T. H. , H. Jensen , H. Pärn , et al. 2015. “On Being the Right Size: Increased Body Size Is Associated With Reduced Telomere Length Under Natural Conditions.” Proceedings of the Biological Sciences 282: 20152331. 26631569 10.1098/rspb.2015.2331 PMC4685786

[emi470373-bib-0051] Rohart, F. , B. Gautier , A. Singh , and K.‐A. L. Cao . 2017. “mixOmics: An R Package for ‘Omics Feature Selection and Multiple Data Integration.” PLoS Computational Biology 13: e1005752. 29099853 10.1371/journal.pcbi.1005752 PMC5687754

[emi470373-bib-0052] Savage, V. M. , J. F. Gillooly , J. H. Brown , G. B. West , and E. L. Charnov . 2004. “Effects of Body Size and Temperature on Population Growth.” American Naturalist 163: 429–441. 10.1086/381872 15026978

[emi470373-bib-0053] Segata, N. , J. Izard , L. Waldron , D. Gevers , L. Miropolsky , and W. S. Garrett . 2011. “Metagenomic Biomarker Discovery and Explanation.” Genome Biology 12: 60. 10.1186/gb-2011-12-6-r60 PMC3218848 21702898

[emi470373-bib-0054] Shapira, M. 2016. “Gut Microbiotas and Host Evolution: Scaling Up Symbiosis.” Trends in Ecology & Evolution 31: 539–549. 27039196 10.1016/j.tree.2016.03.006

[emi470373-bib-0055] Smith, S. N. , T. J. Colston , and C. D. Siler . 2021. “Venomous Snakes Reveal Ecological and Phylogenetic Factors Influencing Variation in Gut and Oral Microbiomes.” Frontiers in Microbiology 12: 657754. 33841384 10.3389/fmicb.2021.657754 PMC8032887

[emi470373-bib-0056] Stevens, C. E. , and I. D. Hume . 1998. “Contributions of Microbes in Vertebrate Gastrointestinal Tract to Production and Conservation of Nutrients.” Physiological Reviews 78: 393–427. 9562034 10.1152/physrev.1998.78.2.393

[emi470373-bib-0057] Tamanai‐Shacoori, Z. , I. Smida , L. Bousarghin , et al. 2017. “ *Roseburia* spp.: A Marker of Health?” Future Microbiology 12: 157–170. 28139139 10.2217/fmb-2016-0130

[emi470373-bib-0058] Tian, Y. , and Y. H. Li . 2017. “Comparative Analysis of Bacteria Associated With Different Mosses by 16S rRNA and 16S rDNA Sequencing.” Journal of Basic Microbiology 57: 57–67. 27515736 10.1002/jobm.201600358

[emi470373-bib-0059] Trevelline, B. K. , K. J. MacLeod , T. Langkilde , and K. D. Kohl . 2019. “Gestation Alters the Gut Microbiota of an Oviparous Lizard.” FEMS Microbiology Ecology 95: fiz086. 31210275 10.1093/femsec/fiz086

[emi470373-bib-0060] Turnbaugh, P. J. , R. E. Ley , M. Hamady , C. M. Fraser‐Liggett , R. Knight , and J. I. Gordon . 2007. “The Human Microbiome Project.” Nature 449: 804–810. 17943116 10.1038/nature06244 PMC3709439

[emi470373-bib-0061] van Best, N. , M. W. Hornef , P. H. M. Savelkoul , and J. Penders . 2015. “On the Origin of Species: Factors Shaping the Establishment of Infant's Gut Microbiota.” Birth Defects Research Part C: Embryo Today: Reviews 105: 240–251. 26607554 10.1002/bdrc.21113

[emi470373-bib-0062] Vasconcelos, D. S. , D. Harris , I. Damas‐Moreira , A. Pereira , and R. Xavier . 2023. “Factors Shaping the Gut Microbiome of Five Species of Lizards From Different Habitats.” PeerJ 11: e15146. 37187519 10.7717/peerj.15146 PMC10178224

[emi470373-bib-0063] Walterson, A. M. , and J. Stavrinides . 2015. “ *Pantoea*: Insights Into a Highly Versatile and Diverse Genus Within the Enterobacteriaceae.” FEMS Microbiology Reviews 39: 968–984. 26109597 10.1093/femsre/fuv027

[emi470373-bib-0064] Wang, S. P. , L. A. Rubio , S. H. Duncan , et al. 2020. “Pivotal Roles for pH, Lactate, and Lactate‐Utilizing Bacteria in the Stability of a Human Colonic Microbial Ecosystem.” mSystems 5: 10.1128/msystems.00645‐20. 10.1128/mSystems.00645-20 PMC7483512 32900872

[emi470373-bib-0065] Zhou, J. , Y.‐T. Zhao , Y. Dai , et al. 2020. “Captivity Affects Diversity, Abundance, and Functional Pathways of Gut Microbiota in the Northern Grass Lizard Takydromus Septentrionalis.” MicrobiologyOpen 9, no. 9: e1095. 32666685 10.1002/mbo3.1095 PMC7520994

