# Supplementary material for: Exploring the Development of Wild Microbiomes in the Eastern Fence Lizard
Source: Environ Microbiol Rep. 2026 Jul 28;18(4):e70373. doi: 10.1111/1758-2229.70373 (PMC13410953; doi:10.1111/1758-2229.70373)
Supplement: Supplementary file 1 — Figure S1: Alpha rarefaction curves for alpha diversity analysis. Figure S2: Classification error plots from PSL‐DA analysis. Table S1: Descriptive statistics for figure 2A measuring observed features. Table S2: Descriptive statistics for figure 2B measuring faith's phylogenetic diversity. Table S3: Alpha diversity values table for observed features Kruskal–Wallis pairwise comparisons. Table S4: Alpha diversity values table for faith's phylogenetic diversity Kruskal–Wallis pairwise comparisons. [file EMI4-18-e70373-s001.docx]

Figure S1. Alpha Rarefaction Curves For Alpha Diversity Analysis


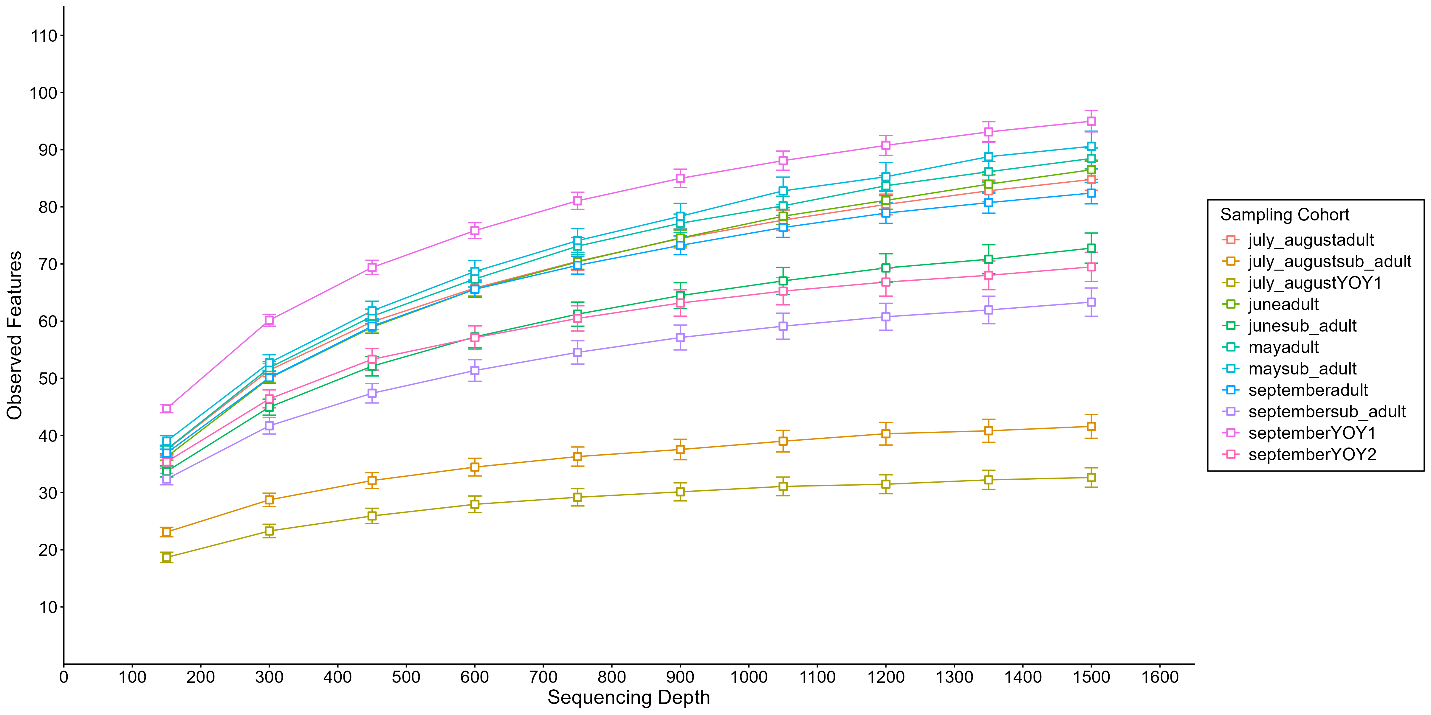


Figure S2. Classification Error Plots From PSL-DA Analysis


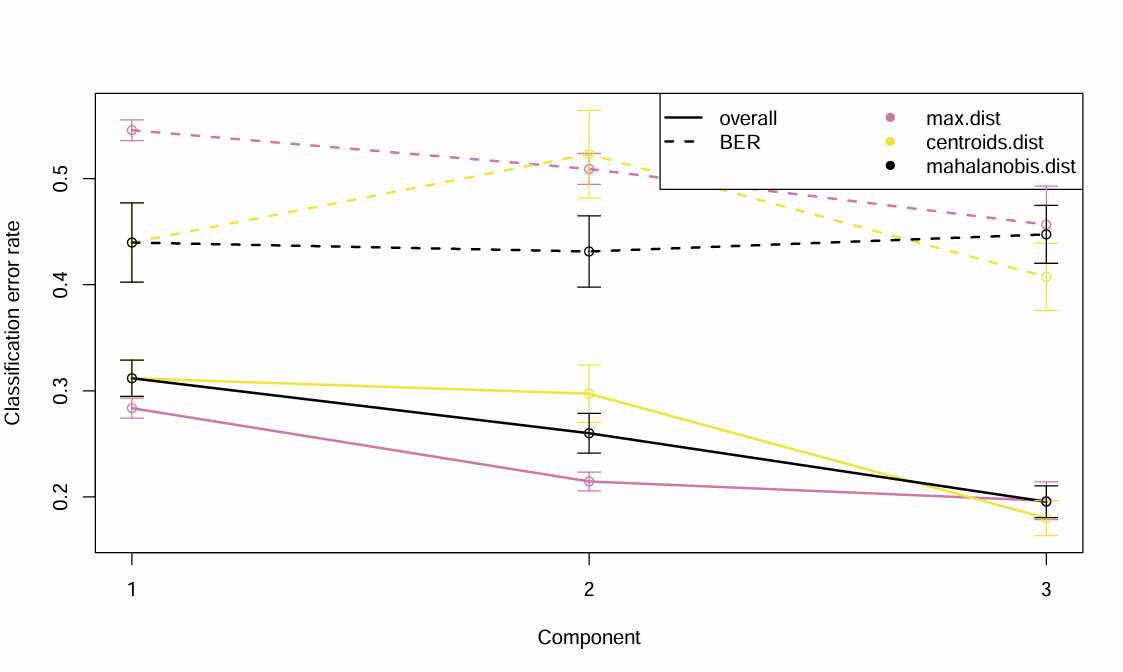


Table S1. Descriptive Statistics for Figure2A Measuring Observed Features

| **Group** | **Median** | **SD** | **Total Features** |
| --- | --- | --- | --- |
| August YOY1 | 21.8 | 25.149232 | 326.5 |
| September YOY2 | 74.475 | 41.094879 | 834 |
| September YOY1 | 101.3 | 29.990772 | 1139.85 |
| May Adults | 94.5 | 42.68263 | 2300 |
| June Adults | 91.05 | 38.172295 | 2162.3 |
| August Adults | 88.25 | 30.320971 | 1017.5 |
| September Adults | 97.05 | 31.339358 | 1071.3 |
| May Rising Adults | 103.4 | 45.711812 | 1268.35 |
| June Rising Adults | 86.15 | 47.037985 | 1091.7 |
| August Rising Adults | 24.275 | 33.451823 | 499.1 |
| September Rising Adults | 61.175 | 39.889919 | 760 |

Table S2. Descriptive Statistics for Figure2B Measuring Faith’s Phylogenetic Diversity

| **Group** | **Median** | **SD** |
| --- | --- | --- |
| August YOY1 | 2.577293849 | 1.706176498 |
| September YOY2 | 5.943138502 | 2.24274209 |
| September YOY1 | 7.246266573 | 1.502320389 |
| May Adults | 6.483508984 | 2.117295059 |
| June Adults | 7.005540137 | 2.115922659 |
| August Adults | 6.690269967 | 1.709431962 |
| September Adults | 7.38175111 | 1.908514881 |
| May Rising Adults | 7.169529232 | 2.331241883 |
| June Rising Adults | 6.002722215 | 2.622566403 |
| August Rising Adults | 2.913527598 | 1.97770728 |
| September Rising Adults | 5.768620938 | 2.358653919 |

Table S3. Alpha Diversity values table for observed features Kruskal-Wallis pairwise comparisons

| **Group 1** | **Group 2** | **H** | **p-value** | **q-value** |
| --- | --- | --- | --- | --- |
| August YOY1 (n=10) | September YOY1 (n=12) | 12.67826087 | 0.0003699 | 0.0163355 |
| August YOY1 (n=10) | August Adult (n=12) | 11.30869565 | 0.0007714 | 0.0163355 |
| August YOY1 (n=10) | June Adult (n=25) | 11.04133333 | 0.000891 | 0.0163355 |
| August YOY1 (n=10) | May Adult (n=26) | 10.32972973 | 0.001309 | 0.0171835 |
| August YOY1 (n=10) | September Adult (n=13) | 10.00384615 | 0.0015621 | 0.0171835 |
| August Rising Adult (n=12) | September YOY1 (n=12) | 9.363333333 | 0.0022137 | 0.0202922 |
| August Adult (n=12) | August Rising Adult (n=12) | 9.013333333 | 0.0026802 | 0.0210585 |
| August YOY1 (n=10) | May Rising Adult (n=14) | 8.571428571 | 0.0034148 | 0.0234767 |
| August Rising Adult (n=12) | June Adult (n=25) | 7.967368421 | 0.0047628 | 0.0291061 |
| August Rising Adult (n=12) | September Adult (n=13) | 7.396449704 | 0.0065353 | 0.035944 |
| August Rising Adult (n=12) | May Adult (n=26) | 7.125246548 | 0.0076006 | 0.038003 |
| August Rising Adult (n=12) | May Rising Adult (n=14) | 5.597883598 | 0.0179822 | 0.0824184 |
| August YOY1 (n=10) | September YOY2 (n=12) | 4.734782609 | 0.0295586 | 0.1250555 |
| August YOY1 (n=10) | September Rising Adult (n=12) | 4.452173913 | 0.0348568 | 0.1369376 |
| August YOY1 (n=10) | June Rising Adult (n=15) | 4.328587918 | 0.0374773 | 0.1374169 |
| September YOY1 (n=12) | September Rising Adult (n=12) | 3 | 0.0832645 | 0.2862218 |
| August Rising Adult (n=12) | September Rising Adult (n=12) | 2.803333333 | 0.0940686 | 0.3043394 |
| August Rising Adult (n=12) | September YOY2 (n=12) | 2.43 | 0.1190329 | 0.3395107 |
| June Adult (n=25) | September Rising Adult (n=12) | 2.325263158 | 0.1272887 | 0.3395107 |
| May Adult (n=26) | September Rising Adult (n=12) | 2.272189349 | 0.1317137 | 0.3395107 |
| September YOY1 (n=12) | September YOY2 (n=12) | 2.253333333 | 0.1333269 | 0.3395107 |
| May Rising Adult (n=14) | September Rising Adult (n=12) | 2.224867725 | 0.1358043 | 0.3395107 |
| August Rising Adult (n=12) | June Rising Adult (n=15) | 2.002380952 | 0.1570523 | 0.3600724 |
| August Adult (n=12) | September Rising Adult (n=12) | 2.001703639 | 0.1571225 | 0.3600724 |
| May Rising Adult (n=14) | September YOY2 (n=12) | 1.523809524 | 0.2170439 | 0.4774966 |
| May Adult (n=26) | September YOY2 (n=12) | 1.424063116 | 0.2327358 | 0.4813511 |
| June Adult (n=25) | September YOY2 (n=12) | 1.402534677 | 0.2362996 | 0.4813511 |
| June Rising Adult (n=15) | May Adult (n=26) | 1.114285714 | 0.2911522 | 0.5545178 |
| September YOY1 (n=12) | September Adult (n=13) | 1.068047337 | 0.3013869 | 0.5545178 |
| June Rising Adult (n=15) | May Rising Adult (n=14) | 1.007619048 | 0.3154739 | 0.5545178 |
| May Rising Adult (n=14) | September Adult (n=13) | 0.989901854 | 0.3197664 | 0.5545178 |
| August YOY1 (n=10) | August Rising Adult (n=12) | 0.97826087 | 0.3226285 | 0.5545178 |
| September Adult (n=13) | September Rising Adult (n=12) | 0.855029586 | 0.3551334 | 0.591889 |
| June Adult (n=25) | June Rising Adult (n=15) | 0.774439024 | 0.3788473 | 0.6128412 |
| August Adult (n=12) | September YOY1 (n=12) | 0.653333333 | 0.4189234 | 0.6583083 |
| August Adult (n=12) | September YOY2 (n=12) | 0.563333333 | 0.4529203 | 0.6900483 |
| June Rising Adult (n=15) | September YOY1 (n=12) | 0.535714286 | 0.4642143 | 0.6900483 |
| September YOY2 (n=12) | September Adult (n=13) | 0.5 | 0.4795001 | 0.6940133 |
| August Adult (n=12) | May Rising Adult (n=14) | 0.447089947 | 0.5037201 | 0.7103746 |
| June Rising Adult (n=15) | September Rising Adult (n=12) | 0.238095238 | 0.6255852 | 0.8414859 |
| May Adult (n=26) | September Adult (n=13) | 0.227218935 | 0.6335937 | 0.8414859 |
| June Adult (n=25) | September YOY1 (n=12) | 0.206315789 | 0.6496705 | 0.8414859 |
| June Adult (n=25) | May Rising Adult (n=14) | 0.192857143 | 0.6605492 | 0.8414859 |
| August Adult (n=12) | June Adult (n=25) | 0.177894737 | 0.6731887 | 0.8414859 |
| August Adult (n=12) | May Adult (n=26) | 0.142011834 | 0.7062891 | 0.8465953 |
| May Adult (n=26) | May Rising Adult (n=14) | 0.125648345 | 0.7229874 | 0.8465953 |
| June Adult (n=25) | September Adult (n=13) | 0.125207101 | 0.7234542 | 0.8465953 |
| September YOY2 (n=12) | September Rising Adult (n=12) | 0.083333333 | 0.77283 | 0.8642145 |
| May Adult (n=26) | September YOY1 (n=12) | 0.079881657 | 0.7774579 | 0.8642145 |
| August Adult (n=12) | September Adult (n=13) | 0.073964497 | 0.7856496 | 0.8642145 |
| August Adult (n=12) | June Rising Adult (n=15) | 0.05952381 | 0.8072502 | 0.8684906 |
| June Adult (n=25) | May Adult (n=26) | 0.05112426 | 0.8211184 | 0.8684906 |
| June Rising Adult (n=15) | September YOY2 (n=12) | 0.038095238 | 0.845252 | 0.8771483 |
| May Rising Adult (n=14) | September YOY1 (n=12) | 0.023809524 | 0.8773706 | 0.8936182 |
| June Rising Adult (n=15) | September Adult (n=13) | 0.013262599 | 0.9083156 | 0.9083156 |

Table S4. Alpha Diversity values table for Faith’s Phylogenetic Diversity Kruskal-Wallis pairwise comparisons

| **Group 1** | **Group 2** | **H** | **p-value** | **q-value** |
| --- | --- | --- | --- | --- |
| August YOY1 (n=10) | September YOY1 (n=12) | 13.15217 | 0.000287 | 0.0157952 |
| August YOY1 (n=10) | August Adult (n=12) | 11.3087 | 0.000771 | 0.0182937 |
| August YOY1 (n=10) | June Adult (n=25) | 10.32533 | 0.001312 | 0.0182937 |
| August YOY1 (n=10) | May Adult (n=26) | 9.659875 | 0.001883 | 0.0182937 |
| August YOY1 (n=10) | September Adult (n=13) | 9.615385 | 0.00193 | 0.0182937 |
| August Rising Adult (n=12) | September YOY1 (n=12) | 9.363333 | 0.002214 | 0.0182937 |
| August YOY1 (n=10) | May Rising Adult (n=14) | 9.270857 | 0.002328 | 0.0182937 |
| August Rising Adult (n=12) | June Adult (n=25) | 7.605263 | 0.00582 | 0.0400112 |
| August Adult (n=12) | August Rising Adult (n=12) | 7.363333 | 0.006657 | 0.04068 |
| August Rising Adult (n=12) | September Adult (n=13) | 6.816568 | 0.009032 | 0.0496738 |
| August Rising Adult (n=12) | May Adult (n=26) | 6.154832 | 0.013105 | 0.0655268 |
| August Rising Adult (n=12) | May Rising Adult (n=14) | 5.357143 | 0.020638 | 0.0888821 |
| August YOY1 (n=10) | September Rising Adult (n=12) | 5.326087 | 0.021009 | 0.0888821 |
| August YOY1 (n=10) | September YOY2 (n=12) | 4.734783 | 0.029559 | 0.116123 |
| September YOY1 (n=12) | September YOY2 (n=12) | 3.63 | 0.056747 | 0.2080717 |
| August YOY1 (n=10) | June Rising Adult (n=15) | 3.350769 | 0.067174 | 0.2309093 |
| August Rising Adult (n=12) | September Rising Adult (n=12) | 3 | 0.083265 | 0.2678769 |
| June Rising Adult (n=15) | September YOY1 (n=12) | 2.916667 | 0.087669 | 0.2678769 |
| September YOY1 (n=12) | September Rising Adult (n=12) | 2.803333 | 0.094069 | 0.2723037 |
| May Rising Adult (n=14) | September YOY2 (n=12) | 2.224868 | 0.135804 | 0.3734618 |
| June Rising Adult (n=15) | May Rising Adult (n=14) | 2.074286 | 0.1498 | 0.3800882 |
| September YOY2 (n=12) | September Adult (n=13) | 2 | 0.157299 | 0.3800882 |
| June Rising Adult (n=15) | September Adult (n=13) | 1.974005 | 0.160023 | 0.3800882 |
| August Rising Adult (n=12) | September YOY2 (n=12) | 1.92 | 0.165857 | 0.3800882 |
| May Adult (n=26) | September YOY1 (n=12) | 1.5 | 0.220671 | 0.485477 |
| September Adult (n=13) | September Rising Adult (n=12) | 1.431953 | 0.231446 | 0.4875614 |
| June Adult (n=25) | September YOY2 (n=12) | 1.364211 | 0.242809 | 0.4875614 |
| August Adult (n=12) | September YOY1 (n=12) | 1.333333 | 0.248213 | 0.4875614 |
| June Adult (n=25) | June Rising Adult (n=15) | 1.280195 | 0.257863 | 0.4890501 |
| June Adult (n=25) | September Rising Adult (n=12) | 1.146316 | 0.284322 | 0.5061671 |
| August YOY1 (n=10) | August Rising Adult (n=12) | 1.113043 | 0.291421 | 0.5061671 |
| August Adult (n=12) | September YOY2 (n=12) | 1.08 | 0.298698 | 0.5061671 |
| June Rising Adult (n=15) | May Adult (n=26) | 1.057875 | 0.3037 | 0.5061671 |
| May Rising Adult (n=14) | September Rising Adult (n=12) | 0.857143 | 0.354539 | 0.5663995 |
| May Adult (n=26) | September YOY2 (n=12) | 0.773176 | 0.379236 | 0.5663995 |
| August Rising Adult (n=12) | June Rising Adult (n=15) | 0.771429 | 0.379775 | 0.5663995 |
| June Adult (n=25) | September YOY1 (n=12) | 0.767368 | 0.381032 | 0.5663995 |
| August Adult (n=12) | June Rising Adult (n=15) | 0.688095 | 0.406813 | 0.5888083 |
| August Adult (n=12) | September Adult (n=13) | 0.579882 | 0.446359 | 0.6294802 |
| August Adult (n=12) | September Rising Adult (n=12) | 0.48 | 0.488422 | 0.6655765 |
| May Adult (n=26) | May Rising Adult (n=14) | 0.463147 | 0.496157 | 0.6655765 |
| May Adult (n=26) | September Rising Adult (n=12) | 0.434911 | 0.509589 | 0.6673188 |
| June Rising Adult (n=15) | September Rising Adult (n=12) | 0.342857 | 0.558185 | 0.7139571 |
| August Adult (n=12) | May Rising Adult (n=14) | 0.26455 | 0.607011 | 0.7587639 |
| May Adult (n=26) | September Adult (n=13) | 0.227219 | 0.633594 | 0.7743922 |
| June Adult (n=25) | May Rising Adult (n=14) | 0.103714 | 0.747417 | 0.8642145 |
| May Rising Adult (n=14) | September YOY1 (n=12) | 0.095238 | 0.757621 | 0.8642145 |
| June Adult (n=25) | September Adult (n=13) | 0.085444 | 0.770051 | 0.8642145 |
| August Adult (n=12) | June Adult (n=25) | 0.085263 | 0.770288 | 0.8642145 |
| September YOY1 (n=12) | September Adult (n=13) | 0.073964 | 0.78565 | 0.8642145 |
| September YOY2 (n=12) | September Rising Adult (n=12) | 0.053333 | 0.817361 | 0.8814681 |
| June Adult (n=25) | May Adult (n=26) | 0.022722 | 0.880183 | 0.9006292 |
| June Rising Adult (n=15) | September YOY2 (n=12) | 0.021429 | 0.883617 | 0.9006292 |
| May Rising Adult (n=14) | September Adult (n=13) | 0.021193 | 0.884254 | 0.9006292 |
| August Adult (n=12) | May Adult (n=26) | 0.008876 | 0.924941 | 0.9249414 |
